# Impact assessment of the WHO Framework Convention on Tobacco Control: introduction, general findings and discussion

**DOI:** 10.1136/tobaccocontrol-2018-054429

**Published:** 2018-09-04

**Authors:** Pekka Puska, Mike Daube

**Affiliations:** 1 National Institute for Health and Welfare, Helsinki, Finland; 2 Curtin University, Perth, Western Australia, Australia

**Keywords:** prevention, public policy, global health, surveillance and monitoring, tobacco industry

## Introduction

The emergence of non-communicable diseases (NCDs) as the leading cause of global death and disease became evident towards the end of the 1990s. Awareness of this development led in 2000 to the adoption of the World Health Organization’s (WHO) Global Strategy on Prevention and Control of Non-communicable Diseases and subsequent publicity.^1 2^

Well before this, governments, health authorities and non-governmental organisations (NGOs) had developed a range of actions in many countries and internationally to combat smoking. There was overwhelming evidence on the devastating and growing impacts of tobacco use on public health. The WHO has long had a commitment to tobacco control: in 1970, the World Health Assembly called on governments to take action to reduce smoking,^3^ and in 1979, a report of the WHO Expert Committee on Smoking Control described a blueprint of various policy interventions to reduce the prevalence of smoking.^4^ With growing international pressure, negotiations were started in the late 1990s to prepare and adopt an international framework convention to curb the global tobacco epidemic. The WHO Framework Convention on Tobacco Control (FCTC) was adopted in 2003 and entered into force in 2005.^5^

Tobacco control became one of the key pillars of the WHO Global NCD Strategy that emphasised prevention through influencing the four main behavioural risk factors: tobacco use, unhealthy diet, physical inactivity and harmful use of alcohol. Indeed, the FCTC was the first specific instrument for this work. It was followed by the WHO Global Strategy on Diet and Physical Activity in 2004 and the Global Strategy on Harmful Use of Alcohol in 2010,^6 7^ but what was and remains unique for tobacco is that the FCTC is an international legal instrument, binding in countries that have ratified it (currently 181 Parties, including the European Union).

## Implementation of the FCTC impact assessment

In the 12 years in which the FCTC has been in effect, much work has been done in participating countries towards tobacco control and adoption of the Treaty’s provisions. The global management of the FCTC was organised via a Secretariat in Geneva, with the roles of supporting and monitoring its development. The countries that have ratified the Convention (the ‘Parties’), meet every 2 years at the Conference of the Parties (COP) to decide on matters including management and further action.

The sixth Meeting of the COP in 2014 in Moscow determined that 10 years after adoption of the FCTC, it was time to carry out an overall impact assessment. The meeting further decided that an independent group of experts should be set up to undertake the task. This independent Expert Group (EG) comprising seven members, reflecting different parts of the world and different types of expertise, was established and carried out the work in 2015–2016. The EG presented its report to the seventh Meeting to the COP in New Delhi in November 2016.^8^

The details of the methodology used by the EG are reported separately in this volume.^9^ In short, the EG used progress reports sent by countries to the Secretariat and available scientific articles, and missions to 12 countries representing different WHO regions and different economic levels. During their visits, the EG interviewed a large number of different stakeholders in the country about the process of tobacco control and specifically about the role of the FCTC. This gave important qualitative information on how and to what extent the FCTC had influenced and contributed to tobacco control in each country.

## Main results

The findings from the Global Evidence Review^10 11^ showed that since the FCTC came into force, there have been significant gains in tobacco control, but with great variability across countries and policy areas. Generally, there has been much progress in the areas of tobacco labelling, education, training and public awareness and restrictions on sales to minors. The FCTC has also contributed to progress in the implementation of measures for tobacco price and tax, advertising and promotion, cessation and surveillance. Many countries have sought to implement the critical Article 5.3 to curb tobacco industry interference with tobacco control efforts, but with varying degree of success.

The EG country missions found strong affirmation of the importance and use of the FCTC and of its Guidelines in providing an agenda for action and a tool for governments to plan and implement their tobacco control work. An important finding was that the FCTC has clearly helped to broaden action from a restricted health focus to recognition of the broader responsibilities of different government sectors in controlling tobacco use. In this context, many countries have created coordinating cross-government and multistakeholder national structures for tobacco control.

The FCTC has also clearly helped NGOs and their coalitions to press governments and to mobilise community support. Article 5.3 has been important in many countries in restricting tobacco industry efforts to undermine tobacco control implementation.

The country visits also confirmed that the tobacco industry appears to have intensified its opposition to tobacco control with even more aggressive approaches, notably through means such as:

- Use of third-party groups and ‘socially responsible’ activities.
- Litigation and other legal measures to oppose and delay tobacco control measures.
- Use of international trade and related agreements to oppose regulatory measures.
- Efforts to present the industry as a partner in tobacco control.

## Conclusions of the impact assessment

The evidence collected by the EG and visits to the countries clearly showed that the FCTC has made a major contribution to tobacco control policy development and implementation. A separate study^12^ also showed that countries that have implemented the FCTC at higher levels have also generally experienced greater reduction in smoking prevalence.

It is clear that the FCTC has provided a roadmap for policies and a catalyst for action for stronger tobacco control. It is apparent that, especially in low-income and middle-income countries that previously had very weak tobacco control, ratification of the FCTC had a major role in supporting the introduction of effective tobacco control. It is, however, also noteworthy that even in developed countries with many previous tobacco control measures, the Convention has clearly helped to strengthen tobacco control.

The FCTC has helped countries in legal defenses against the tobacco industry^13^ and increased awareness of tobacco industry interference.^14^ The FCTC has also been instrumental in strengthening international collaboration and linkages between countries and international agencies. In this it has spearheaded stronger international NCD work.

## General discussion

Few doubt the need for effective global NCD prevention and the central role of tobacco control in this work. The creation of the FCTC in 2003 was undoubtedly a landmark, as a unique international health Convention, and as a practical and legal measure supporting this approach.

Thus, the question of the impact of the FCTC is of great interest. To prove the causal role of the FCTC is not an easy task. There is no possibility for an experimental study design. With growing evidence of the harmful role of tobacco on the disease burden, much progress would also undoubtedly have taken place even without the Convention, although opposed, resisted and undermined by the global tobacco industry.

Country reports and other data show the trends and provide other information on smoking and tobacco use during the 10 years of the Convention. These show some reductions in smoking prevalence over time and a greater reduction in countries with stronger implementation.

Two comments are obvious. First, the FCTC was introduced during the time when smoking was still increasing in most low-income and middle-income countries. Thus, even slowing this increase could be an effect. Second, the fact that countries with stronger implementation of FCTC provisions have shown a greater reduction does not necessarily fully prove the impact of the Convention.

In our view, the strongest evidence on the role of the FCTC came from the qualitative data in the country visits. During those visits, for several days and in different kinds of countries, the members of the EG interviewed a great number of stakeholders from Government and other organisations, to ask about the specific role of the FCTC in tobacco control in each country. After this, the EG had no doubt that the FCTC has in numerous countries been a strong catalyst for action, a guide for evidence-based measures, and a strong support for withstanding the lobbying and other influences of the tobacco industry.

The EG of course noted also the many obstacles hindering progress and commented on those in its report. By far, the greatest obstacle is the aggressive approach of the tobacco industry, which directly opposes and undermines the FCTC and its recommendations. Further important obstacles include inertia within governments and the very limited resources available for tobacco control in both governments and the community.

In its report, the EG made several recommendations to enhance the process and to strengthen the impact of the FCTC. These include stronger adherence to Article 5.3 (which was seen as the single highest priority), more effective use of tax measures, better surveillance and more international expert and resource support.

Since tobacco is a major risk factor for many NCDs, it is properly one of the four behavioural targets for NCD prevention in the overall WHO NCD Strategy. As work to implement the strategy has subsequently been developed for the Global WHO Action Plan on NCD Prevention and Control 2013–2020, specific targets have been adopted to arrive at a 25% reduction in premature NCD mortality by 2025.

For tobacco, the target adopted was a 30% reduction. Even if in many developed countries this (taken from 2010) is feasible, the target for the world as a whole is very ambitious. On the other hand, it has been estimated that the reduction in tobacco use is crucial to reach the 25% NCD reduction.^15^

After adoption of the WHO NCD Action Plan, the United Nations has agreed on Global Sustainable Development Goals.^16^ These goals also confirm the role of health and well-being in global goals of sustainable development and to strengthen the global NCD target of a 30% reduction by 2030. A major reduction in tobacco use is a prime measure for sustainable development, with substantial health gains achieved in a sustainable way, but also with other consequences linked to sustainable development.

Through implementation of measures recommended in the FCTC, tobacco control in many countries has also shown the way for ‘Health in All Policies’ approaches.^17^ This is in part because FCTC implementation work has broadened the range of activity entailed, and involved many other sectors than health, which had previously been seen as bearing the sole responsibility.

Given positive experience from the FCTC, there have at times been suggestions for other conventions as stronger ‘hard instruments’ to enhance work on NCDs. As the Impact Assessment shows, a Convention is not an easy instrument. But the FCTC Impact Assessment has given valuable insight to the many aspects of a health-related Convention.

The emergence of widespread tobacco use with its catastrophic consequences is in many ways linked with issues of globalisation. The International Labour Organization Commission on ‘Social consequences of globalisation’ noted in its 2004 report that in addition to many favourable impacts, globalisation also has negative effects on people and environments. The Commission recommended that the international community should take action to counteract these negative consequences.^18^ The FCTC is a prime example of determined and hard action to counteract a global health problem.

## Conclusion

The EG was clear in concluding that the FCTC had played an important role in curbing the global tobacco epidemic and countering the activities of the tobacco industry. At the same time, the findings described in greater detail in this issue provide many lessons that it is hoped will help to strengthen the implementation of the Convention in countries and globally, and thus spearhead global work for NCD prevention and sustainable development.

## References

[1] World Health Organization. Global strategy for the prevention and control of noncommunicable diseases. Report by the Director General. EB105/42. 2000 http://apps.who.int/iris/handle/10665/78986

[2] World Health Organization & Public Health Agency of Canada. Preventing chronic diseases—a vital investment. 2005 http://apps.who.int/iris/bitstream/handle/10665/43314/9241563001_eng.pdf?sequence=1

[3] World Health Organization. WHA23.32 Health consequences of smoking. 1970 http://www.who.int/tobacco/framework/wha_eb/wha23_32/en/

[4] WHO Expert Committee on Smoking Control & World Health Organization. Controlling the smoking epidemic. Report of the WHO expert Committee on Smoking Control. World Health Organ Tech Rep Ser 1979:7–87. 112784

[5] World Health Organization. WHO Framework Convention on Tobacco Control. 2003 http://apps.who.int/iris/bitstream/10665/42811/1/9241591013.pdf?ua=1

[6] World Health Organization. Global plan for prevention and control of NCDs 2013–2020. 2013 http://www.who.int/nmh/events/ncd_action_plan/en/

[7] World Health Organization. Global strategy to reduce the harmful use of alcohol. 2010 http://www.who.int/substance_abuse/activities/gsrhua/en/ 10.2471/BLT.19.241737 PMC7047030 32132758

[8] WHO Convention Secretariat. Impact Assessment of the WHO FCTC. 2016 http://www.who.int/fctc/implementation/impact/en/

[9] Fong GT, Chung-Hall J, Craig L WHO FCTC Impact Assessment Expert Group. Impact assessment of the WHO FCTC over its first decade: methodology of the expert group. Tob Control 2019;28(suppl 2):s84–s88. 10.1136/tobaccocontrol-2018-054374 29954859 PMC6589466

[10] Chung-Hall J, Craig L, Gravely S, et al Impact of the WHO FCTC over the first decade: a global evidence review prepared for the Impact Assessment Expert Group. Tob Control 2019;28(suppl 2):s119–s128. 10.1136/tobaccocontrol-2018-054389 29880598 PMC6589489

[11] Chung-Hall J, Craig L, Gravely S, et al Impact of the WHO Framework Convention on Tobacco Control on the implementation and effectiveness of tobacco control measures: a global evidence review. 2016 http://www.who.int/fctc/cop/cop7/Documentation-Supplementary-information/en/

[12] Gravely S, Giovino GA, Craig L, et al Implementation of key demand-reduction measures of the WHO Framework Convention on Tobacco Control and change in smoking prevalence in 126 countries: an association study. Lancet Public Health 2017;2:e166–e174. 10.1016/S2468-2667(17)30045-2 29253448

[13] Zhou SY, Liberman JD, Ricafort E The impact of the WHO Framework Convention on Tobacco Control in defending legal challenges to tobacco control measures. Tob Control 2019;28(suppl 2):s113–s118. 10.1136/tobaccocontrol-2018-054329 29860233 PMC6589463

[14] Bialous S Impact of implementation of the WHO FCTC on the tobacco industry’s behaviour. 2015 http://www.who.int/fctc/cop/cop7/Documentation-Supplementary-information/en/ 10.1136/tobaccocontrol-2018-054808 PMC6589456 30659104

[15] Beaglehole R, Bonita R, Horton R, et al Priority actions for the non-communicable disease crisis. Lancet 2011;377:1438–47. 10.1016/S0140-6736(11)60393-0 21474174

[16] United Nations. Transforming our world: the 2030 agend for sustainable development. A/RES/70/1. 2015 https://sustainabledevelopment.un.org/content/documents/21252030%20Agenda%20for%20Sustainable%20Development%20web.pdf

[17] Puska P, Ståhl T Health in all policies—the Finnish initiative: background, principles, and current issues. Annu Rev Public Health 2010;31:315–28. 10.1146/annurev.publhealth.012809.103658 20070201

[18] World Commission on the Social Dimension of Globalization. A fair globalization: creating opportunities for all. 2004 http://www.ilo.org/public/english/wcsdg/docs/report.pdf

